# Vegetation structure and photosynthesis respond rapidly to restoration in young coastal fens

**DOI:** 10.1002/ece3.2348

**Published:** 2016-09-07

**Authors:** Anna M. Laine, Anne Tolvanen, Lauri Mehtätalo, Eeva‐Stiina Tuittila

**Affiliations:** ^1^ Department of Forest Science University of Helsinki P.O. Box 27 FI‐00014 Helsinki Finland; ^2^ Department of Ecology University of Oulu P.O. Box 3000 FI‐90014 Oulu Finland; ^3^ Natural Resources Institute Finland (Luke) University of Oulu P.O. Box 413 FI‐90014 Oulu Finland; ^4^ School of Computing University of Eastern Finland P.O. Box 111 FI‐80101 Joensuu Finland; ^5^ School of Forest Sciences University of Eastern Finland P.O. Box 111 FI‐80101 Joensuu Finland; ^6^ Present address: Department of Ecology University of Oulu P.O. Box 3000 FI‐90014 Oulu Finland

**Keywords:** CO_2_, forestry drainage, low resistance, peatland restoration, plant functional type, resilience, species composition, succession

## Abstract

Young coastal fens are rare ecosystems in the first stages of peatland succession. Their drainage compromises their successional development toward future carbon (C) reservoirs. We present the first study on the success of hydrological restoration of young fens. We carried out vegetation surveys at six young fens that represent undrained, drained, and restored management categories in the Finnish land uplift coast before and after restoration. We measured plant level carbon dioxide (CO_2_) assimilation and chlorophyll fluorescence (Fv/Fm) from 17 most common plant species present at the sites. Within 5 years of restoration, the vegetation composition of restored sites had started to move toward the undrained baseline. The cover of sedges increased the most in response to restoration, while the cover of deciduous shrubs decreased the most. The rapid response indicates high resilience and low resistance of young fen ecosystems toward changes in hydrology. Forbs had higher photosynthetic and respiration rates than sedges, deciduous shrubs, and grasses, whereas rates were lowest for evergreen shrubs and mosses. The impact of management category on CO_2_ assimilation was an indirect consequence that occurred through changes in plant species composition: Increase in sedge cover following restoration also increased the potential photosynthetic capacity of the ecosystem. *Synthesis and applications*. Restoration of forestry drained young fens is a promising method for safeguarding them and bringing back their function as C reservoirs. However, their low resistance to water table draw down introduces a risk that regeneration may be partially hindered by the heavy drainage in the surrounding landscape. Therefore, restoration success is best safeguarded by managing the whole catchments instead of carrying out small‐scale projects.

## Introduction

While primary succession has been a common form of peatland initiation after deglaciation, northern peatland succession series are currently globally rare. They can presently be found only in three regions with ongoing land uplift: at the coasts of Bothnia Bay in Finland and Sweden, coast of White Sea in Russia, and in the Hudson Bay Lowlands of Canada. Young coastal fens are the first stages of primary peatland succession and occur in locations where frequent floods influence the vegetation (Klinger and Short 1996). In the beginning of the succession, large temporal variation in water table is characteristic due to the shallow peat layer, low water holding capacity of the sandy soil, and lack of established vegetation typical of later successional states (Leppälä et al. 2008; Tuittila et al. 2013). Waterlogged conditions support accumulation of organic matter as peat and while succession proceeds, mire hydrology is decreasingly controlled by allogenic factors such as precipitation, surface inflow and runoff, and variation in sea level. This autogenic succession proceeds from minerotrophic, groundwater‐fed fens toward ombrotrophic bogs (Hughes and Dumayne‐Peaty 2002; Rydin and Jeglum 2013).

During succession, resistance increases and resilience decreases (Odum 1969). Resistant systems are able to maintain a given state when they are subjected to disturbances. Resilient systems are sensitive to disturbances, that is, they respond by changing their structure and functions, but recover rapidly toward their original state when the source of disturbance is removed (Allison and Martiny 2008). Previous studies on the impacts of forestry drainage and restoration show that peatlands tend to conform to the theory of Odum (1969): The nutrient‐poor, late‐successional bogs appear to be resistant systems with slow and often partial secondary succession following drainage (Laine et al. 1995), and slow regeneration after restoration (Jauhiainen et al. 2002; Laine et al. 2011). In contrast, young fens that lack the buffering thick peat layer typical to bogs can be expected to have low resistance to hydrological disturbances.

In Finland, the occurrence of young coastal fens is defined to a zone <20 m a.s.l. (Rehell and Heikkilä 2009). Their succession starts similarly to, for example, European dune slacks, where the bare mineral soil is colonized by pioneer species that are adapted to temporarily or permanently waterlogged conditions (Grootjans et al. 1998). Young coastal fens covered some 74,000 ha but are strongly impacted by land management and approximately 95% have been drained (rough estimates based on Rehell and Heikkilä 2009). The percentage is considerably higher than the average peatland drainage percentage, 50%, of the original area of 10.4 million hectares in Finland (Finnish Statistical Yearbook of Forestry 2014). Intensive drainage is the main reason that peatland succession series are considered critically endangered (Rehell and Heikkilä 2009).

Peatland drainage for forestry, which impacts approximately 15 million hectares of peatlands globally (Paavilainen and Päivänen 1995), alters the hydrological regime, increases the aeration of peat, and redirects the development toward forest succession (Laine et al. 1995; Vompersky and Sirin 1997; Mälson et al. 2008). The landscape diversity decreases due to the replacement of peatland plant species by species common in the surrounding forests (Vasander 1987). Changes in the species composition may influence ecosystem functions, such as photosynthesis (e.g., Reich et al. 1998), biomass production, and litter decomposability (Laiho 2006). For example, increased shading by trees and lowered water table may directly influence the rate of photosynthesis (Medrano et al. 2003). The increased peat respiration rate lowers the C accumulation capacity (Minkkinen et al. 2007) that is a key ecosystem service of peatlands. In young fens, drainage prohibits the C accumulation that would otherwise occur during the successional peatland development.

Restoration aims to assist the recovery of the structure and function of damaged ecosystems toward that of pristine ecosystems (e.g., Hobbs and Cramer 2008). In global and national policies, it is currently seen as a crucial means to safeguard biodiversity (Aichi Biodiversity Targets 2011; EU Biodiversity Strategy to 2020). The principal restoration methods for forestry drained boreal peatlands are the blocking of ditches to recreate the high water table and the removal of excess trees to reduce the transpiration rate and reinstate the landscape typical of natural peatlands (e.g., Tarvainen et al. 2013). The high water table can be quickly restored and common peatland species, such as *Sphagnum* mosses and sedges may rapidly recolonize the sites (e.g., Haapalehto et al. 2011). However, specialist‐species, such as hepatics, rare rich fen indicators, and hollow/flark species may still be missing from restored sites several years after restoration (Hedberg et al. 2012; Maanavilja et al. 2014). The desired species and plant functional type composition depends on the peatland type. Young fens are typically dominated by sedges, grasses, and forbs, and their moss layer is scattered (Leppälä et al. 2008). Hence, high amounts of mosses or shrubs are not expected after restoration, as for bogs.

Because restoration of young fens has not been implemented to date, little information exists on the regeneration of the structure and function of these ecosystems. Knowledge gained from so‐called true peatlands with thick peat layers may not be applicable to young fen ecosystems due to differences in peat thickness, plant species composition, nutrient dynamics, and hydrology, which may crucially influence peatland responses to drainage and restoration.

This is the first study to quantify the impacts of forestry drainage and restoration on water table, vegetation composition, and functioning in terms of CO_2_ assimilation in the rare young fen ecosystems. We hypothesize that (1) due to the low resistance of young fens to disturbance, drainage has changed their hydrology and ecosystem functions, and directed their successional pathway toward forest; (2) as a consequence of the low resistance, rewetting with felling redirects their vegetation composition toward that of undrained fens already during the first years after restoration, that is, favors sedges, grasses, and forbs; and (3) the expected change in vegetation is reflected as increased photosynthetic rates while shrubs that dominate in drained peatlands are replaced by graminoids and forbs with higher photosynthetic capacity.

## Material and Methods

### Study area

The study was carried out in the Finnish land uplift coast of the Gulf of Bothnia in Siikajoki (64°48′N, 24°38′E). The 30 years (1979–2009) average precipitation and mean annual temperature are 539 mm and 2.6°C, respectively, and the length of growing season is 150 days (Revonlahti, Siikajoki, 64° 41′N, 25° 05′E, 48 m a.s.l., Finnish Meteorological Institute). We selected six coastal fens belonging to three management categories in 2005: two undrained (UD1, UD2), two drained (D1, D2), and two drained sites to be restored later (R1, R2). The sites represent the same predrainage coastal young fen type that was formed by primary mire development following postglacial land uplift approximately 100–200 years ago (Ekman 1996). They are located in small depressions of ~0.5–3 ha between nutrient‐poor sand dunes. The sites lie at 1.5–2 m asl and have a 5‐ to 10‐cm‐thick organic soil layer. The topography of the area is diverse and undulating. Finding exactly similar study sites was therefore not possible.

The two undrained sites are located in a small nature protection area ~4 km from the drained and restored sites that are located approximately 700 m apart. The plant community of the undrained sites is composed of graminoids and forbs. Moss layer is scattered and bare soil forms a major part of the ground (Table S1).

In drained state, the ground layer vegetation was mainly formed by shrubs although at D2 and R2 sedges were also abundant. The tree stand volume of the two drained sites was 71 and 21 m^3^ (D1, D2, respectively), and in restoration sites, it was 87 and 10 m^3^ (R1, R2, respectively). Restoration of the sites R1 and R2 was carried out in two phases in 2008. First, between 75% and 100% of the stems were felled in spring, and 0–5 trees left per 100 m^2^. The tree stand densities corresponded to those before drainage that were estimated using aerial photos from the 1960s. Second, ditches were blocked using excavators in August 2008.

### Water table and vegetation composition measurements

Water table (WT) was measured from perforated plastic tubes, 3 cm in diameter, drilled into 50 cm depth in the ground. In each site, there were 3–6 additional 100‐cm‐long tubes that were used to correct the measurements during extreme droughts. In the drained and restored sites, WT measurements were carried out in May, August, and October from 2005 to 2014 and in the undrained sites in 2007 and in 2010–2014. Vegetation was recorded at 12–14 permanent sample plots of 1 × 1 m in the restored and drained sites 2 years before restoration in 2006 and 1 and 5 years after restoration in 2009 and 2013, respectively. For the undrained sites, vegetation was recorded at six 0.6 × 0.6 m sample plots in 2007, 2010, and 2013. The undrained sites were part of another research project, which explains their different sampling protocol. The spatial variation of the vegetation was low at the undrained sites, which allowed their lower number of sample plots. Percentage covers of plants were defined visually by consensus of two experienced researchers working together, using the scale 0.25%, 0.5%, 1%, 2%, 3%, etc. We used quadrat frames, divided into four 25% segments by strings, to assist the observation.

### CO_2_ assimilation and chlorophyll fluorescence measurements

To quantify the light response of photosynthesis, CO_2_ assimilation (A) was measured in July 2011, with a portable open, fully controlled, flow through gas exchange fluorescence measurement system (GFS‐3000; Walz, Germany) under varying light levels. To account for the direct effects of drainage and restoration, we selected common plant species occurring in all sites so that the effect on the same species can be compared between sites. To study the indirect effect, that is, effect caused by changing plant species composition due to management, we chose the most common species within each site (Table S2). Six samples per species per management category (undrained, drained, and restored) were measured. Half of the samples were collected within open‐top chambers (OTC) located in each site for a study of global warming impacts. As the OTC treatment had no impact on CO_2_ assimilation rates (tested with general linear models, see below), those samples were included in this study. The device was set up nearby the study sites, and plants were picked at maximum 30 min before the measurement with an ample amount of roots and soil, then kept moist and in shaded conditions. Depending on the species, we enclosed one or several leaves within the cuvette. A standard leaf cuvette was used for all species other than shrubs and mosses, for which the conifer cuvette was used. CO_2_ assimilation was measured at 800, 50, 20, and 0 μmol m^−2^ sec^−1^ photosynthetic photon flux density (PPFD). The sample was allowed to adjust to cuvette conditions for 5 min before the first measurement and then for 3 min after each change in the PPFD level; otherwise, the cuvette conditions were kept constant (temperature 20°C, CO_2_ concentration 380 ppm, relative humidity 60%, flow rate 400, and impeller in level 5). The time required for a full measurement cycle was 20 min.

Each sample was stored in a paper bag and transported to the laboratory, where they were stored in a cold room until the dry weight was measured after drying at 105°C for at least 12 h. We calculated CO_2_ assimilation per dry mass (*μ*mol g DM^−1^ h^−1^).

The intrinsic quantum efficiency of PSII (Fv/Fm) was measured with a portable fluorometer (FMS‐2; Hansatech, King's Lynn, UK) from intact samples on site. This allowed measurement of more species and a higher sample size (Table S2). Leaves were dark acclimated with leaf clips (Hansatech) for at least 20 min before the measurement.

To quantify the species specific chlorophyll pigment concentrations (Ensminger et al. 2001), six leaf samples from all plant species used in CO_2_ assimilation measurements were collected and frozen in liquid nitrogen immediately in field, and stored at −80°C for 4 months. Prior to analysis, leaf samples (~50 mg) were ground in liquid nitrogen, freeze dried, and extracted with 100% acetone buffered with sodium bicarbonate (NaHCO_3_) for 2 h at 4°C. Pigment concentrations in the extracts were determined using a spectrophotometer (Shimadzu UV‐1700, Kyoto, Japan) in the laboratory of the Department of Biology, University of Oulu.

### Data analysis

We applied detrended correspondence analysis (DCA) to reveal the main community gradients within the data set and to visualize the temporal movement of communities within management categories over the gradients/ordination space. The temporal changes in different management categories were compared using principal response curves (PRC). In the analysis, the measuring years for unmanaged sites were considered similar to those for managed sites. This method is a derivative of redundancy analysis (RDA) that focuses on the differences between the species compositions of the treatments at each sampling date. It allows the time trajectory of species composition in the control treatment to be displayed as a horizontal line against which deviations in species composition under other experimental treatments can be plotted. The abundance of each species is modeled as a sum of three terms: the species' mean abundance in the control, a date‐specific treatment effect, and an error (Poulin et al. 2013). The analyses were performed with Canoco 5.01 for Windows.

To further analyze the impact of the management category on vegetation, plant species were pooled to nine plant functional types (PFT): sedges, grasses, forbs, evergreen shrubs, deciduous shrubs, *Sphagnum*, forest mosses, mire mosses, and liverworts (Table S1). For each PFT, we tested the effect of management, measuring year (2006/2007, 2009/2010, 2013), their interaction, and WT on the cover of the PFT with a linear mixed model. The full model for each PFT was

(1) $$y_{ijk}=T_{i}+YR_{k}+T_{i}*YR_{k}+wt_{ijk}+a_{i}+b_{ij}+e_{ijk}$$

where *y*_*ijk*_ is the transformed cover of the PFT in year *k* of sample plot *j* on site *i, T*_*i*_ is the fixed effect of management, *YR*_k_ is the fixed effect of year *k*,* T*_i_*YR_*k*_, is the interaction of management and year, and *wt*_*ijk*_ is the fixed effect of water table. Terms *a*_*i*_ and *b*_*ij*_ are normally distributed, zero‐mean random effects for site and plot within site, and *e*_*ijk*_ is the residual error. The transformation for *y*_*ijk*_ was an arcsin transformation of a power‐transformed cover, where the power was determined for each response so that the model showed a constant variance with no clear trends in the mean of residuals. Water table was removed from the models when it was statistically insignificant and negatively impacted the model performance, using conditional *F*‐tests with critical *P* value of 0.05. Once the final model was found, post hoc conditional *t*‐tests were carried out to compare the differences between management categories in 2006/2007 and 2013 using both undrained and drained as a control.

To determine the effects of PFT, management, and chlorophyll content on the light response parameters of net photosynthesis, we applied a nonlinear mixed‐effects model with the hyperbolic light saturation curve (e.g., Lappi and Oker‐Blom 1992):

(2) $$A_{ksi}=R_{ks}+\frac{P_{{\mathrm{MAX}}_{ks}}{\text{PPED}}_{ksi}}{\alpha +{\text{PPFD}}_{ksi}}+e_{ksi}$$

where the response *A*_*ksi*_ is the observed net photosynthesis expressed on a dry weight basis, and the predictor PPFD_*ksi*_ is the photosynthetic photon flux density for measurement *i* of sample *s* on site *k*. The parameters to be estimated are respiration (*R*_*ks*_), photosynthetic capacity, that is, the maximum rate of light‐saturated gross photosynthesis ($P_{{\mathrm{MAX}}_{ks}}$), and the maximum quantum yield of CO_2_ assimilation (*α*), that is, light use efficiency at low light. The residual (*e*_*ksi*_) is normally distributed with mean zero and constant variance. In the full model, parameters *α, R*_*ks,*_ and $P_{{\mathrm{MAX}}_{ks}}$ were written as linear functions of fixed predictors PFT, management category and chlorophyll content, and random effects for nested levels of site and sample within site. These linear submodels were included in the light saturation curve (2), and all coefficients were estimated in one step. However, the full model was not estimable, and model fitting included fitting different models. After an estimable model was found, tests on the different effects were conducted using approximate conditional *F*‐tests (*P* > 0.05). For this analysis, we re‐grouped the four moss PFTs into two PFTs by including *Sphagnum fimbriatum* in mire mosses (Table S2). Sedge PFT and undrained category were used as the standard with which other PFTs and management categories were compared. Final submodels for the photosynthesis parameters in equation (2) were

(3) $$R_{ks}={\text{PFT}}_{ks}+{\text{Treat}}_{ks}+\text{chl}+a_{ks}$$

(4) $$P_{{\mathrm{MAX}}_{ks}}={\text{PFT}}_{ks}+{\text{Treat}}_{ks}+\text{chl}+b_{k}+b_{ks}$$

(5) $$\alpha_{ks}={\text{PFT}}_{ks}+{\text{Treat}}_{ks}+\text{chl}+c_{ks}$$

where PFT_*ks*_, Treat_*ks*_, and chl are effects for PFT (seven levels), management category (three levels), and chlorophyll *a* + *b* content (continuous), respectively. Terms *a*_*ks*_, *b*_*ks*_, *c*_*ks*_ are trivariate normal random effects for sample*s* on site *k*, and *b*_*k*_ is univariate normal random effect for site. The random effects take into account variability in *P*_MAX_ and *α* that was not explained by the fixed effects, and ensures that the conducted tests take into account the lack of independence caused by the grouped structure of the data. Nested random effects were originally included in all three models at two nested levels (site and sample), but only those shown to be significant were included in the final models. For sedges, forbs, and combined mosses, which occurred in all management categories, we tested the interaction between PFT and management treatment for the *P*_MAX_ parameter.

To determine the effects of PFT (Table S2) and management on Fv/Fm ratio, we used a linear mixed‐effects model (1), using the fifth power of Fv/Fm as the response variable.

All models were fitted, and the tests were performed using package nlme of the R software, following the procedures of Pinheiro and Bates (2000, Chapter 8).

## Results

### Water table

**Figure 1:**
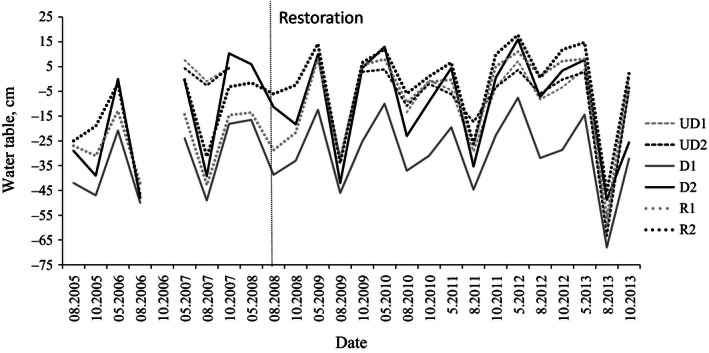
Average water table depth (cm) in the six study sites measured in May, August, and October in 2005–2013. Negative values indicate water table level below the soil surface. UD denotes for undrained, D for drained, and R for restored sites. Restoration by ditch blocking and clear cutting was carried out in 2008. The depth of the peat layer is <10 cm; therefore, most of the variation on water table occurs in the mineral soil substratum.

Water table was highly variable both spatially and temporally (Fig. 1). Before restoration, the WT was on average 20 cm lower at the drained/restored categories than at undrained category. The differences between the two sites within each management category were high: Sites D1 and R1 were drier with on average 12 cm lower WT before restoration than sites D2 and R2, respectively. The difference in WT between the drier site D1 and the wetter site D2 was on average 19 cm during the whole study period. During the springs before restoration, sites D2 and R2 had WT close to or at surface, while in D1 and R1 WT stayed below the surface. Following restoration, the WT levels at sites R1 and R2 became similar to those in the undrained sites, and the average difference between them decreased to 3 cm. Seasonal variation was high: Site D2 and all undrained and restored sites experienced spring and autumn floods. During a very dry period in 2013, the WT dropped to an extremely low level at all sites (Fig. 1).

### Vegetation composition

**Figure 2:**
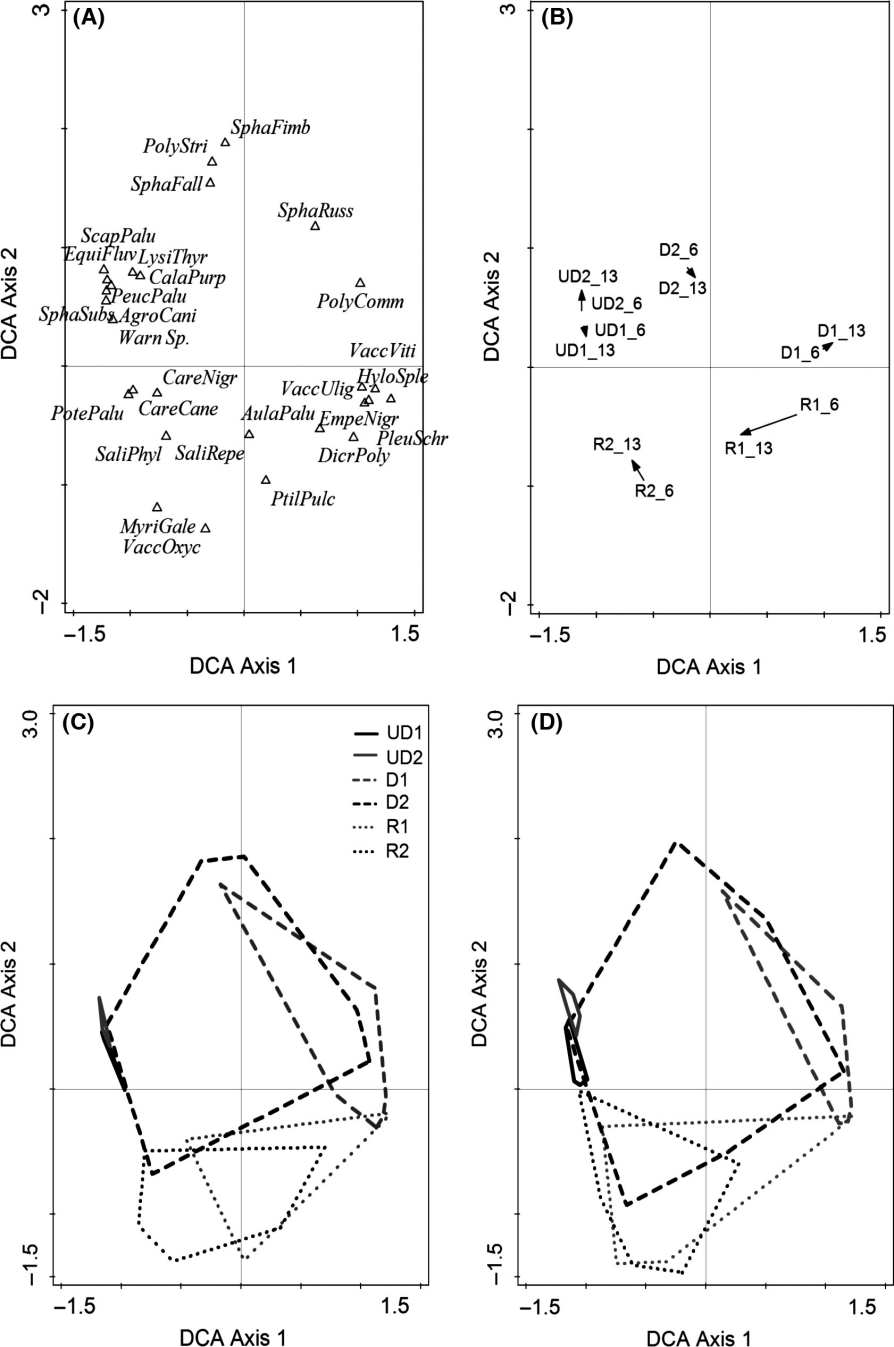
Detrended correspondence analysis (DCA) for the vegetation composition data 2006 (before restoration) and 2013 (5 years after restoration). Eigenvalues of the first and second axes are 0.868 and 0.626, respectively. The axes together explained 24% of the variation in the data. (A) Species that have at least 5% fit on both axes (28 of 50 species); (B) movement of study site centers between 2006 and 2013, the length of the arrow indicates the amount of movement, (C) enveloped sample plots of each site in 2006, and (D) enveloped sample plots of each site in 2013. Species full names are given in Table S1.

**Figure 3:**
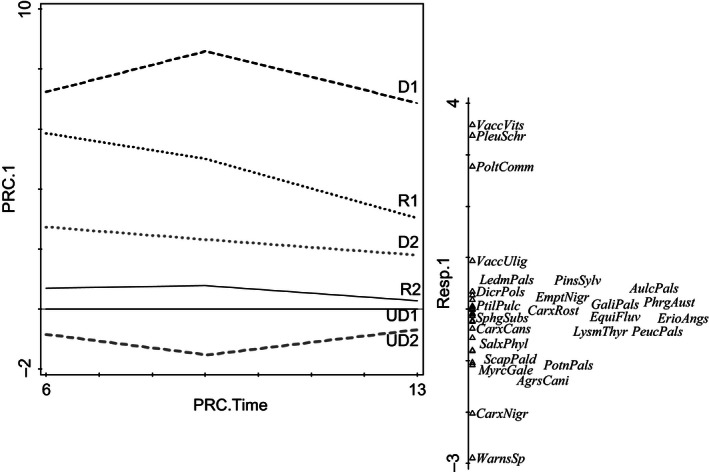
Principal response curve (PRC) analysis, with undrained site 1 (UD1) as reference; effect of management/site, including its interaction with time, is significant according to Monte Carlo permutation test (*F* = 33.4, *P* = 0.002). UD = undrained, D = drained, and R = restored. Dashed vertical line indicates the year of restoration (time point 0). The line on the right shows the species scores for the first ordination axis. Fen species from undrained sites are at the bottom end. Forest species are at the top.

Fifty plant species were recorded in the six study sites. According to DCA, the main vegetation gradient was related to the drainage succession where mesic wetland species were replaced by forest species (left to right along the first DCA axis) (Fig. 2A). Two years before restoration in 2006, the vegetation composition of the four drained sites was variable and differed from that of the undrained category (Fig. 2B and C). In addition, vegetation heterogeneity was much greater in the drained and restored categories than in the undrained category. Five years after restoration, the vegetation composition of many sample plots had moved toward the composition of the undrained category (Fig. 2B and D). PRC analysis confirmed this result: All management categories originally differed from each other, but the restored category was more similar to the undrained category and further approached the undrained category within 5 years of restoration (Fig. 3). The vegetation of the drained category also moved toward undrained category, especially at regularly flooded site D2, but this change was more modest than in restored category (Fig. 3). Five new plant species were recorded after restoration in 2013 at the originally wetter site R2 (Table S1). These were typical species for fens: *Calamagrostis purpurea*,* Eriophorum angustifolium, Calliergon cordifolia, Sphagnum fimbriatum,* and *S. squarrosum*. Four moss species had disappeared: *Straminergon stramineum, Dicranum polysetum, Pohlia nutans, and Polytrichum strictum*. At site R1 five new species typical to fens: *E. angustifolium, Carex rostrata, Potentilla palustris, Calliergon cordifolia,* and *Sphagnum russowii* and three forest moss species: *Dicranum fuscescens, Polytrichum commune,* and *Ptilidium ciliare* were observed after restoration, while none of the species had disappeared. Seven fen species were found at the undrained sites but not in the restored sites: *Agrostis canina, Alnus glutinosa, Equisetum fluviatile, Lysimachia thyrsiflora, Peucedanum palustre*,* Sphagnum fallax,* and *S. subsecundum* (Table S1).

### Impact of management on plant functional types

**Figure 4:**
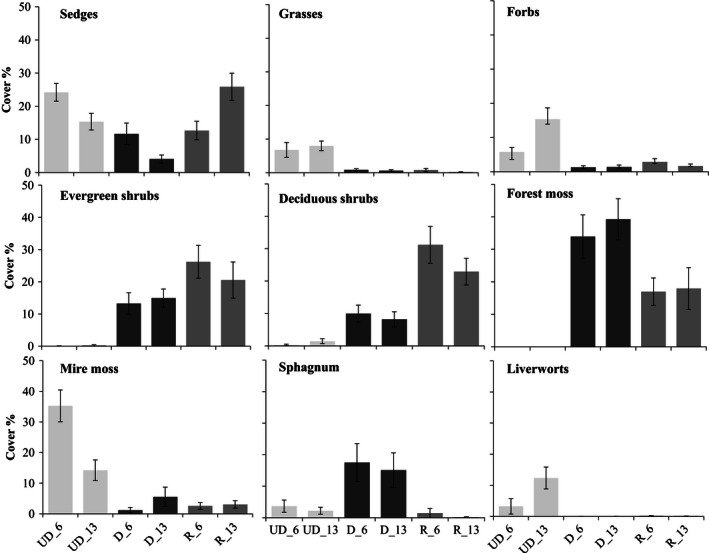
Cover ± SE of PFTs in management categories (UD = undrained, D = drained, and R = restored) before restoration in 2006 and 5 years after restoration in 2013.

We found a significant management impact on sedges and grasses (*P* < 0.05, Table S3). Prior to restoration, the cover of deciduous shrubs was higher and the cover of grasses lowers in the restored category than in the undrained category. The cover of grasses and brown mosses was lower in the drained category than in the undrained category. Possibly due to low number of replicates, there were no statistically significant differences between the restored and drained categories (Table S4), although the cover of shrubs seemed to be greater, and that of forest mosses and *Sphagnum* lower in the restored category (Fig. 4).

The cover of PFTs differed between the three study years, with the exception of the evergreen shrubs, forest mosses, and *Sphagnum*, which did not show any change (Fig. 4, Table S3). The direction of change, that is, the increase or decrease in cover, was different between the management categories (Fig. 4), which was seen as the significant interaction between management and year for all other PFTs than forest mosses and *Sphagnum*. (Table S3). Five years after restoration, the sedge cover of the restored category had increased to a higher level than that at the undrained and drained categories (Fig. 4). Deciduous shrub cover had decreased at the restored category and was no longer significantly higher than at the undrained category (Table S4). After inclusion of management category, the impact of WT was not significant for the cover of any PFT (Table S3).

### CO_2_ exchange of plant functional types

**Figure 5:**
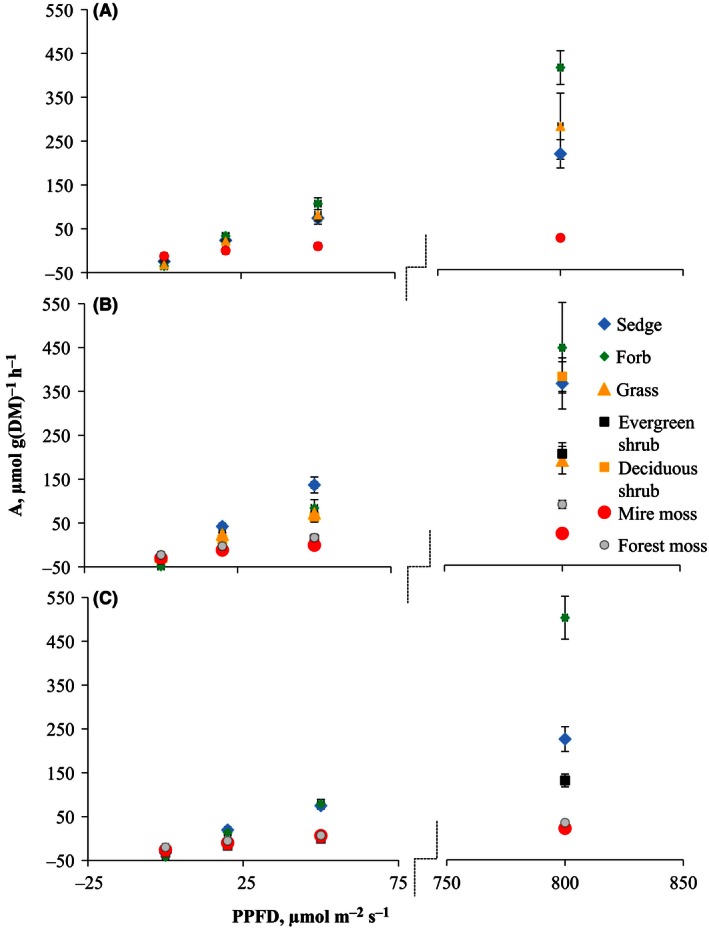
Average carbon dioxide (CO_2_) assimilation of different plant functional types measured at photosynthetic photon flux density (PPFD) 0, 20, 50, and 800 *μ*mol m^−2^ sec^−1^ in (A) undrained, (B) drained, and (C) restored categories. Breakage of *x*‐axis between PPFD 75 and 750.

Maximal photosynthesis (*P*_MAX_) varied between PFTs. Forbs had significantly higher, and evergreen shrubs, mire mosses, and forest mosses lower *P*_MAX_ than had sedges (Fig. 5, Table S5 Model 1, Table S6). Sedges were chosen as the standard for the models as they occur in all study sites. Respiration rate (R) varied between PFTs and management categories (Table S5). Forbs had significantly higher and mosses significantly lower R values than sedges (Fig. 5, Table S6), and R was higher at the restored and drained categories than at the undrained category. The maximum quantum yield of CO_2_ assimilation (*α*) depended on PFT (Table S5). It was higher for forbs, deciduous, and evergreen shrubs than for sedges, and lower for mire mosses than for sedges (Fig. 5, Table S6). Chlorophyll *a* + *b* content, which varied between PFTs (Table S2), had a significant effect only for R (Table S5, Model 2).

After testing the whole range of PFTs across the study areas, we focused on the direct effects of management on sedges, forbs and combined forest and mire mosses, which occurred in all management categories. All estimated parameters from Equation (2) (*P*_MAX_, *R*, and *α*) were significantly affected by PFT and management category so that that plants growing on drained category had significantly higher *P*_MAX_ values than those on undrained category (Model 3 in Tables S5 and S6). Similarly to Model 1, forbs had higher and mosses lower *P*_MAX_ values compared to sedges (Table S6). In addition, we found an interaction between management category and PFT (Table S6), so that mosses on drained category had significantly lower *P*_MAX_ values than was generally typical of mosses. Similarly to Model 1, respiration rate (*R*) was higher (i.e., more negative) at both restored and drained categories than at undrained category (Table S6, Model 3), and forbs had higher and mosses lower respiration rates than sedges. The maximum quantum yield of CO_2_ assimilation (*α*) was higher in both the restored and drained categories (*P* value = 0.063) than in the undrained category, and forbs had higher and combined mosses lower *α* than sedges (*P* value = 0.081) (Table S6, Model 3).

### The intrinsic quantum efficiency of PSII (Fv/Fm)

**Figure 6:**
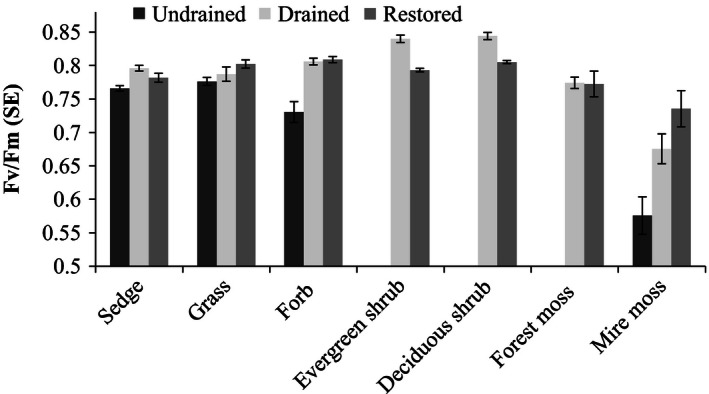
The intrinsic quantum efficiency of PSII (Fv/Fm) measured for seven PFTs management types: undrained, drained, and restored.

The measured Fv/Fm ratio varied between 0.21 and 0.87. Shrubs and grasses had significantly higher and mire mosses significantly lower Fv/Fm ratios than sedges (Fig. 6, Table S7), whereas there were no differences in Fv/Fm between management categories.

## Discussion

The allogenic control of hydrology and succession in young coastal fens is somewhat comparable to central European dune slacks. In dune slacks, interannual variation in the water table, and especially the winter flooding, are the most important factors determining vegetation succession (Grootjans et al. 1998). This phenomenon can also be observed in young coastal fens. Unlike in dune slacks however, during mire development, the autogenic control of water table gains a level that is able to support peat forming vegetation and allows mire succession to proceed after the connection to the flooding sea is lost (Tuittila et al. 2007; Leppälä et al. 2011).

As the landscape where young coastal fens are located in Finland is intensively drained, their succession is largely disturbed. There is no earlier information on how these ecosystems response to environmental perturbations such as draining and restoration. Although coastal fens are special ecosystems, the knowledge attained from them can be used to assess the outcome of environmental perturbations in analogous, allogenically controlled ecosystems. They serve as ecological laboratories where the outcome of environmental changes can be tested and rapidly seen.

### Drainage impacts in young fens

As proposed in our first hypothesis, the hydrology, ecosystem functions, and vegetation composition had changed as a consequence of drainage. The change that was observed in the successional pathway within three decades is generally observed also on older forestry drained peatlands but over longer time scale (Laine et al. 1995). The relatively rapid change indicates that young fens have low resistance to disturbances. However, because these ecosystems are located in flood‐prone locations close to the sea and do not have buffering peat deposits, drainage efficiency varied with local topography. This resulted into a range of microhabitats where some were similar to those found in undrained fens and others more suited for forest species. If the wetter sites such as D2 and R2 continue to experience regular spring and autumn floods, the efficiency of drainage is compromised by large water table fluctuations.

### Effect of restoration on vegetation

As proposed in the second hypothesis, the successional development of young fens was redirected toward that of undrained fens during the first years after restoration. Nevertheless, local variation in hydrology seemed to regulate the postrestoration succession as it had regulated the postdrainage succession. This was demonstrated by the flood‐prone site R2 becoming more similar to the undrained sites, despite the faster vegetation change at the drier site R1. Sedges were the first plants to respond to higher water table and increased light availability that followed tree felling. Sedges respond rapidly to restoration also in older forestry drained peatlands and in cut away peatlands (Komulainen et al. 1999; Tuittila et al. 2000; Graf et al. 2008). The most common species in our sites, *Carex nigra* and *C. canescens,* are tolerant to a wide range of water table conditions including flooding (Visser et al. 2000). These two species had persisted at all sites throughout the drainage period. Availability of propagules of the desired species is crucial to ensure the desired species composition (Pärtel et al. 1998). The small number of new species that had established after restoration and the fact that only seven species found in undrained sites were not present in restored sites suggest that the young fen species pool is high even after three decades of drainage. In older forestry drained peatlands, vegetation recovery requires a considerably longer time period than the 5 years of our study, although the response is faster in nutrient‐rich fens than at nutrient‐poor sites (Haapalehto et al. 2011; Laine et al. 2011; Hedberg et al. 2012).

### Effect of restoration on photosynthesis

As proposed by our third hypothesis, the changes in species composition were reflected as increased photosynthetic rates. Peatland management affects ecosystem functions largely through water table changes (e.g., Laiho 2006; Waddington et al. 2010), and management effects on photosynthetic performance are mainly indirect. As observed earlier (e.g., Reich et al. 1998) and in this study, chlorophyll concentration and the rates of photosynthesis and respiration are lower in evergreen shrubs and mosses than in sedges and forbs. The two latter PFTs dominated in our undrained sites, while shrubs were more common in drained sites. It is notable that the cover of sedges increased after restoration, which enhances ecosystem‐level photosynthesis. Thus, by favouring plant groups with high photosynthetic capacity, restoration of young fens is likely to bring back their important capacity to store some of the captured carbon. In addition to indirect management effect, there was a direct effect in that sedges, forbs, and mosses at drained sites had higher photosynthesis and respiration rates than the same PFTs at undrained sites. This may have been caused by the rather hot and dry weather during the measurement campaign. Plants growing under shaded conditions, as at the drained sites in our study, may experience less water deficiency during hot weather and hence photosynthesize more vigorously (Hájek et al. 2009). This suggestion is supported by the low Fv/Fm values observed for most PFTs at the undrained sites in our study, although the differences were not significant between drained and restored sites.

## Conclusions

There is potential to safeguard the globally rare young coastal fen ecosystems and bring back their function as potential C reservoirs through restoration as a result of their low resistance to environmental changes. The restoration success is increased, if activities are carried out at the state when regular flooding still occurs due to the low topography and the closeness of the sea. As the autogenic control of young fens is still missing, catchment scale restoration, or restoration of sufficiently large hydrological entities should be favoured to minimize the drainage impact from the surrounding area. This is a challenge, however, as the landscape where young fens are located at least in Finland is under intensive land use.

## Conflict of Interest

None declared.

## Supporting information


**Table S1.** Occurrence of species in study sites.

**Table S2.** Plant species used for carbon dioxide (CO_2_) assimilation and chlorophyll fluorescence (Fv/Fm) measurements.

**Table S3.** Impact of drainage and restoration on the cover of plant functional types (PFTs), ANOVA results.

**Table S4.** Impact of drainage and restoration on the cover of plant functional types (PFTs), parameter estimates.

**Table S5.** Impact of drainage and restoration on the light response of photosynthesis, ANOVA results.

**Table S6.** Impact of drainage and restoration on the light response of photosynthesis, Parameter estimates.

**Table S7.** Parameter estimates from the chlorophyll fluorescence (Fv/Fm) model.
