# Supplementary material for: Vegetation structure and photosynthesis respond rapidly to restoration in young coastal fens
Source: Ecol Evol. 2016 Sep 7;6(19):6880–91. doi: 10.1002/ece3.2348 (PMC5513228; doi:10.1002/ece3.2348)
Supplement: Supplementary file 1 — Table S1. Occurrence of species in study sites. [file ECE3-6-6880-s001.docx]

Table S1. Occurrence of species in study sites in years 2006 and 2013. PFT indicates into which plant functional type species is classified. For each site and year, the three dominant vascular plants and mosses are indicated by * and ^, respectively

|  |  | 2006 |  |  |  |  |  | 2013 |  |  |  |  |  |
| --- | --- | --- | --- | --- | --- | --- | --- | --- | --- | --- | --- | --- | --- |
| Species | PFT | R1 | R2 | D1 | D2 | UD1 | UD2 | R1 | R2 | D1 | D2 | UD1 | UD2 |
| *Agrostis canina* | Grass |  |  |  |  | x* | x* |  |  | x | x | x | x* |
| *Alnus glutinosa* | Deci shrub |  |  |  |  | x |  |  |  |  |  | x |  |
| *Aulacomnium palustre* | Forest moss | x | x^ | x |  |  |  | x | x | x |  |  |  |
| *Betula pubescens* | Deci shrub | x | x | x | x |  | x | x | x | x | x | x | x |
| *Brachytecium sp.* | Mire moss |  |  |  |  |  |  |  |  | x |  |  |  |
| *Calamagrostis purpurea* | Grass |  |  |  | x | x | x |  | x |  | x |  |  |
| *Calamagrostis stricta* | Grass |  | x | x | x |  |  |  | x | x | x |  |  |
| *Calliergon cordifolia* | Mire moss |  |  |  |  | x^ |  | x | x^ |  | x | x^ |  |
| *Calliergon stramineum* | Mire moss |  | x |  | x |  |  |  |  |  |  |  |  |
| *Carex acuta* | Sedge |  |  |  | x |  |  |  |  |  | x |  |  |
| *Carex canescens* | Sedge | x | x | x | x | x* | x | x | x* |  | x | x | x |
| *Carex chordorrhiza* | Sedge |  |  |  | x |  |  |  |  |  | x |  |  |
| *Carex magellanica* | Sedge |  | x |  | x |  |  |  | x |  | x |  |  |
| *Carex nigra* | Sedge | x* | x* | x | x* | x* | x* | x* | x* | x | x* | x* | x* |
| *Carex rostrata* | Sedge |  |  | x |  |  |  | x |  |  |  |  |  |
| *Ceratodon purpureus* | Liverwort |  | x |  |  |  |  |  |  |  |  |  |  |
| *Cladina rangiferina* | Lichen |  |  | x |  |  |  | x |  | x |  |  |  |
| *Dicranum fuscescens* | Forest moss |  | x |  |  |  |  | x | x | x | x |  |  |
| *Dicranum majus* | Forest moss |  |  |  |  |  |  |  |  | x |  |  |  |
| *Dicranum polysetum* | Forest moss | x^ | x | x |  |  |  | x^ |  | x | x |  |  |
| *Empetrum nigrum* | Ever shrub | x | x | x |  |  |  | x | x | x | x |  |  |
| *Epilobium palustre* | Forb |  |  |  |  |  |  |  |  |  |  | x |  |
| *Equisetum fluviatile* | Forb |  |  |  |  |  | x |  |  |  |  |  | x |
| *Eriophorum angustifolium* | Sedge |  |  |  | x | x | x | x | x |  | x |  | x |
| *Galium palustre* | Forb |  |  |  | x | x |  |  |  |  |  |  |  |
| *Gymnocolea sp.* | Liverwort |  |  |  |  |  |  |  | x |  |  |  |  |
| *Helodium blandowii* | Mire moss |  |  |  |  |  |  |  |  |  |  |  |  |
| *Hylocomium splendens* | Forest moss | x^ |  |  |  |  |  | x^ |  | x |  |  |  |
| *Juncus filiformis* | Sedge |  |  |  | x* |  |  |  |  |  | x |  |  |
| *Ledum palustre* | Ever shrub |  |  | x* |  |  |  |  |  | x* |  |  |  |
| *Lysimachia thyrsiflora* | Forb |  |  |  | x | x | x |  |  |  | x | x | x |
| *Melampryum pratense* | Forb | x |  |  | x |  |  | x |  |  |  |  |  |
| *Myrica gale* | Deci shrub | x* | x* |  |  |  |  | x* | x* |  |  |  |  |
| *Peucedanum palustre* | Forb |  |  |  |  | x | x |  |  |  |  | x* | x |
| *Phragmites australis* | Grass |  | x |  |  |  |  |  | x |  |  |  |  |
| *Pinus sylvestris* | Ever shrub | x | x | x | x | x | x | x | x | x | x | x |  |
| *Pleurozium schreberi* | Forest moss | x^ | x^ | x^ | x |  |  | x^ | x^ | x^ | x |  |  |
| *Pohlia nutans* | Forest moss | x | x | x |  |  |  | x |  | x |  |  |  |
| *Polytrichum commune* | Forest moss |  |  | x^ | x^ |  |  | x |  | x^ | x^ |  |  |
| *Polytrichum strictum* | Forest moss | x | x |  | x^ |  |  | x |  |  | x |  |  |
| *Potentilla palustris* | Forb |  | x |  | x |  | x* | x | x |  | x | x* | x* |
| *Ptilidium pulcherrimum* | Liverwort |  | x | x |  |  |  | x | x | x |  |  |  |
| *Salix phylicifolia* | Deci shrub | x | x |  | x* | x |  | x | x |  | x* | x |  |
| *Salix repens* | Deci shrub | x | x | x | x |  |  | x | x | x | x |  |  |
| *Sanionia uncinata* | Forest moss |  |  |  |  |  |  |  |  |  |  |  |  |
| *Scapania paludicola* | Liverwort |  | x |  |  |  | x^ |  | x | x |  | x^ | x^ |
| *Sphagnum fallax* | Sphagnum |  |  |  |  |  |  |  |  |  | x |  | x |
| *Sphagnum fimbriatum* | Sphagnum | x |  | x^ | x^ |  |  | x | x | x^ | x^ |  | x^ |
| *Sphagnum russowii* | Sphagnum |  |  |  | x |  |  | x |  | x | x |  |  |
| *Sphagnum squarrosum* | Sphagnum |  |  |  | x |  |  |  | x | x | x | x | x |
| *Sphagnum subsecundum* | Sphagnum |  |  |  | x |  | x^ |  |  |  | x |  | x |
| *Sphagnum teres* | Sphagnum |  |  |  |  |  |  |  |  |  | x |  |  |
| *Vaccinium oxycoccos* | Ever shrub | x | x* | x | x |  |  | x | x | x | x |  |  |
| *Vaccinium uliginosum* | Deci shrub | x | x | x* | x |  |  | x | x | x* | x |  |  |
| *Vaccinium vitis-idaea* | Ever shrub | x* | x | x* | x |  |  | x* | x | x* | x* |  |  |
| *Warnstorfia sp.* | Mire moss | x | x^ |  | x | x^ | x^ | x | x^ |  | x^ | x^ | x^ |
