# Supplementary material for: Vegetation structure and photosynthesis respond rapidly to restoration in young coastal fens
Source: Ecol Evol. 2016 Sep 7;6(19):6880–91. doi: 10.1002/ece3.2348 (PMC5513228; doi:10.1002/ece3.2348)
Supplement: Supplementary file 2 — Table S2. Plant species used for carbon dioxide (CO2) assimilation and chlorophyll fluorescence (Fv/Fm) measurements. [file ECE3-6-6880-s002.docx]

Table S2. Plant species used for carbon dioxide (CO_2_) assimilation and chlorophyll fluorescence (Fv/Fm) measurements. Plant functional type (PFT) and division into upland forest and mesic fen/wetland species for each species; site and management category (UD1, UD2 = undrained; D1, D2 = drained; R1, R2 = restored) for location where species were collected. Average ±SE for specific leaf area (SLA, mm^2^ mg^-1^) and chlorophyll a+b content (µg Chl/mg FW) are given for those vascular plant species used for CO_2_ assimilation measurements.

| Species | PFT | wetland / forest | Management | CO_2_ | Fv/Fm | SLA | Chl a+b |
| --- | --- | --- | --- | --- | --- | --- | --- |
| *Agrostis canina* | Grass | wetland | UD1, UD2 | x | x | 17.4 ±3.2 | 1.3 ±0.13 |
| *Calamagrostis purpurea* | Grass | wetland | D2 | x | x | 15.0± 1.5 | 1.3 ±0.10 |
| *Carex canescens* | Sedge | wetland | UD1, UD2, D2, R2 |  | x |  |  |
| *Carex magellanica* | Sedge | wetland | R2 |  | x |  |  |
| *Carex nigra* | Sedge | wetland | all | x | x | 17.7± 2.9 | 1.2± 0.12 |
| *Eriophorum angustifolium* | Sedge | wetland | UD1, UD2, R2 | x | x | 10.4± 0.4 | 1.1 ±0.06 |
| *Lysimachia thyrsiflora* | Forb | wetland | UD2, D2 |  | x |  |  |
| *Myrica gale* | Deci shrub | wetland | R1 |  | x |  |  |
| *Peucedanum palustre* | Forb | wetland | UD1 |  | x |  |  |
| *Potentilla palustris* | Forb | wetland | UD1, UD2, D2, R2 | x | x | 17.3 ±1.0 | 1.8 ±0.09 |
| *Vaccinium uliginosum* | Deci shrub | forest | D1 | x | D1, R1 | 19.9± 2.4 | 2.6 ±0.36 |
| *Vaccinium vitis-idaea* | Ever shrub | forest | R1, D1 | x | x | 9.2 ±0.6 | 1.7 ±0.36 |
| *Pleurozium schreberi* | ForestMoss | forest | 2,3 | x | x |  | 0.5 ±0.17 |
| *Polytrichum commune* | ForestMoss | forest | D1 | x | x |  | 1.0 ±0.06 |
| *Polytrichum strictum* | ForestMoss | forest | D2 | x | x |  | 0.5 ±0.17 |
| *Sphagnum fimbriatum* | MireMoss | wetland | D2 | x | x |  | 0.2 ±0.04 |
| *Warnstorfia sp.* | MireMoss | wetland | UD1, UD2, D2, R2 | x | x |  | 0.3± 0.06 |
