# Supplementary material for: Vegetation structure and photosynthesis respond rapidly to restoration in young coastal fens
Source: Ecol Evol. 2016 Sep 7;6(19):6880–91. doi: 10.1002/ece3.2348 (PMC5513228; doi:10.1002/ece3.2348)
Supplement: Supplementary file 3 — Table S3. Impact of drainage and restoration on the cover of plant functional types (PFTs), ANOVA results. [file ECE3-6-6880-s003.docx]

Table S3. Impact of drainage and restoration on the cover of plant functional types (PFTs). ANOVA results of the linear mixed effects models for the differences between management category (Management, factorial; undrained, drained, restored), years (factorial: 06, 09, 13), their interaction (Yr: Manage) and water table (WT, continuous) for each PFT. WT was omitted from models if it was not significant and weakened the model.

| PFT | Source | numDF | denDF | F-value | p-value |
| --- | --- | --- | --- | --- | --- |
| Sedge | Intercept | 1 | 122 | 120.27 | <.0001 |
|  | Year | 2 | 3 | 15.37 | 0.0265 |
|  | Management | 2 | 122 | 4.23 | 0.0168 |
|  | Yr: Manage | 4 | 122 | 3.01 | 0.0209 |
| Grass | Intercept | 1 | 122 | 31.17 | <.0001 |
|  | Year | 2 | 3 | 11.44 | 0.0395 |
|  | Management | 2 | 122 | 24.48 | <.0001 |
|  | Yr: Manage | 4 | 122 | 8.49 | <.0001 |
| Forbs | Intercept | 1 | 122 | 14.91 | 0.0002 |
|  | Year | 2 | 122 | 8.59 | 0.0003 |
|  | Management | 2 | 3 | 2.73 | 0.211 |
|  | Yr: Manage | 4 | 122 | 7.54 | <.0001 |
| Deciduous Shrub | Intercept | 1 | 122 | 18.59 | <.0001 |
|  | Year | 2 | 122 | 3.57 | 0.0312 |
|  | Management | 2 | 3 | 3.97 | 0.1437 |
|  | Yr: Manage | 4 | 122 | 4.02 | 0.0043 |
| Evergreen Shrub | Intercept | 1 | 122 | 7.48 | 0.0072 |
|  | Year | 2 | 122 | 1.98 | 0.1427 |
|  | Management | 2 | 3 | 1.67 | 0.3248 |
|  | Yr: Manage | 4 | 122 | 3.24 | 0.0145 |
| Liverworts | Intercept | 1 | 121 | 15.07 | 0.0002 |
|  | Year | 2 | 121 | 8.59 | 0.0003 |
|  | Management | 2 | 3 | 3.28 | 0.176 |
|  | wt | 1 | 121 | 0.58 | 0.447 |
|  | Yr: Manage | 4 | 121 | 3.41 | 0.0111 |
| Miremoss | Intercept | 1 | 122 | 19.16 | <.0001 |
|  | Year | 2 | 122 | 8.36 | 0.0004 |
|  | Management | 2 | 3 | 4.18 | 0.1356 |
|  | Yr: Manage | 4 | 122 | 2.81 | 0.0284 |
| Forestmoss | Intercept | 1 | 121 | 0.00 | 0.9542 |
|  | Year | 2 | 121 | 0.06 | 0.9421 |
|  | Management | 2 | 3 | 1.72 | 0.3175 |
|  | wt | 1 | 121 | 0.25 | 0.6164 |
|  | Yr: Manage | 4 | 121 | 0.20 | 0.9366 |
| *Sphagnum* | Intercept | 1 | 121 | 1.20 | 0.2762 |
|  | Year | 2 | 121 | 0.23 | 0.7962 |
|  | Management | 2 | 3 | 1.32 | 0.3884 |
|  | wt | 1 | 121 | 0.07 | 0.7973 |
|  | Yr: Manage | 4 | 121 | 0.05 | 0.9952 |
