# Supplementary material for: Vegetation structure and photosynthesis respond rapidly to restoration in young coastal fens
Source: Ecol Evol. 2016 Sep 7;6(19):6880–91. doi: 10.1002/ece3.2348 (PMC5513228; doi:10.1002/ece3.2348)
Supplement: Supplementary file 4 — Table S4. Impact of drainage and restoration on the cover of plant functional types (PFTs), parameter estimates. [file ECE3-6-6880-s004.docx]

Table S4. Impact of drainage and restoration on the cover of plant functional types (PFTs). Parameter estimates from linear mixed models concerning the effects of management category before (2006) and after (2013) restoration. The % cover estimates are arcsine transformed with varying power for different PFTs (see in PFT coloum: ^1^asin((cover/100)^(1/4); ^2^asin((cover/100)^(1/3); ^3^asin((cover/100)^(1/2)).

| **Year 2006** | | | | | | | | | |
| --- | --- | --- | --- | --- | --- | --- | --- | --- | --- |
| Contrast Undrained | Intercept |  |  | Drained |  |  | Restored |  |  |
| PFT | Value | Std.Error | DF | Value | Std.Error | DF | Value | Std.Error | DF |
| Sedge^1^ | **0.78** | 0.21 | 122 | -0.41 | 0.29 | 3 | -0.24 | 0.29 | 3 |
| Grass^2^ | **0.40** | 0.07 | 122 | -0.31 | 0.10 | 3 | -0.30 | 0.10 | 3 |
| Forb^3^ | **0.20** | 0.08 | 122 | -0.14 | 0.11 | 3 | -0.09 | 0.11 | 3 |
| EverSh^3^ | 0.02 | 0.17 | 122 | 0.28 | 0.23 | 3 | 0.43 | 0.23 | 3 |
| DeciSh^3^ | 0.02 | 0.12 | 122 | 0.24 | 0.16 | 3 | 0.51 | 0.16 | 3 |
| MireM^3^ | **0.81** | 0.16 | 122 | **-0.73** | 0.22 | 3 | -0.61 | 0.22 | 3 |
| Liverw^1^ | 0.19 | 0.14 | 121 | -0.20 | 0.20 | 3 | -0.15 | 0.20 | 3 |
| Spha^3^ | 0.13 | 0.11 | 121 | 0.15 | 0.16 | 3 | -0.09 | 0.16 | 3 |
| ForeM^3^ | -0.01 | 0.21 | 121 | 0.55 | 0.29 | 3 | 0.30 | 0.29 | 3 |
| Contrast Drained | Intercept |  |  | Undrained |  |  | Restored |  |  |
| PFT | Value | Std.Error | DF | Value | Std.Error | DF | Value | Std.Error | DF |
| Sedge^1^ | 0.37 | 0.21 | 122 | 0.41 | 0.29 | 3 | 0.17 | 0.29 | 3 |
| Grass^2^ | 0.09 | 0.07 | 122 | 0.31 | 0.10 | 3 | 0.01 | 0.10 | 3 |
| Forb^3^ | 0.07 | 0.07 | 122 | 0.14 | 0.11 | 3 | 0.04 | 0.10 | 3 |
| EverSh^3^ | 0.29 | 0.16 | 122 | -0.28 | 0.23 | 3 | 0.15 | 0.23 | 3 |
| DeciSh^3^ | **0.26** | 0.11 | 122 | -0.24 | 0.16 | 3 | 0.27 | 0.16 | 3 |
| MireM^3^ | 0.08 | 0.15 | 122 | **0.73** | 0.22 | 3 | 0.12 | 0.21 | 3 |
| Liverw^1^ | -0.01 | 0.15 | 121 | 0.20 | 0.20 | 3 | 0.06 | 0.20 | 3 |
| **Year 2013** | | | | | | | | | |
| Contrast Undrained | Intercept |  |  | Drained |  |  | Restored |  |  |
| PFT | Value | Std.Error | DF | Value | Std.Error | DF | Value | Std.Error | DF |
| Sedge^1^ | 0.66 | 0.12 | 122 | -0.39 | 0.17 | 3 | 0.06 | 0.17 | 3 |
| Grass^2^ | **0.43** | 0.07 | 122 | **-0.35** | 0.10 | 3 | **-0.40** | 0.10 | 3 |
| Forb^3^ | 0.37 | 0.08 | 122 | -0.31 | 0.11 | 3 | -0.29 | 0.11 | 3 |
| EverSh^3^ | 0.01 | 0.17 | 122 | 0.32 | 0.23 | 3 | 0.34 | 0.23 | 3 |
| DeciSh^3^ | 0.07 | 0.12 | 122 | 0.16 | 0.16 | 3 | 0.39 | 0.16 | 3 |
| MireM^3^ | **0.62** | 0.16 | 122 | -0.45 | 0.22 | 3 | -0.36 | 0.22 | 3 |
| Liverw^1^ | **0.48** | 0.15 | 121 | -0.49 | 0.20 | 3 | -0.44 | 0.20 | 3 |
| Contrast Drained | Intercept |  |  | Undrained |  |  | Restored |  |  |
| PFT | Value | Std.Error | DF | Value | Std.Error | DF | Value | Std.Error | DF |
| Sedge^1^ | 0.27 | 0.11 | 122 | 0.39 | 0.17 | 3 | **0.45** | 0.16 | 3 |
| Grass^2^ | 0.08 | 0.07 | 122 | **0.35** | 0.10 | 3 | -0.05 | 0.10 | 3 |
| Forb^3^ | 0.06 | 0.07 | 122 | 0.31 | 0.11 | 3 | 0.02 | 0.10 | 3 |
| EverSh^3^ | **0.33** | 0.16 | 122 | -0.32 | 0.23 | 3 | 0.02 | 0.23 | 3 |
| DeciSh^3^ | **0.23** | 0.11 | 122 | -0.16 | 0.16 | 3 | 0.22 | 0.16 | 3 |
| MireM^3^ | 0.17 | 0.15 | 122 | 0.45 | 0.22 | 3 | 0.09 | 0.21 | 3 |
| Liverw^1^ | 0.00 | 0.15 | 121 | 0.49 | 0.20 | 3 | 0.05 | 0.20 | 3 |
| Bolded values are significant with p<0.05 and underlined values have p<0.06) | | | | | | | | | |
