# Supplementary material for: Vegetation structure and photosynthesis respond rapidly to restoration in young coastal fens
Source: Ecol Evol. 2016 Sep 7;6(19):6880–91. doi: 10.1002/ece3.2348 (PMC5513228; doi:10.1002/ece3.2348)
Supplement: Supplementary file 5 — Table S5. Impact of drainage and restoration on the light response of photosynthesis, ANOVA results. [file ECE3-6-6880-s005.docx]

Table S5. Impact of drainage and restoration on the light response of photosynthesis. ANOVA results of the hyperbolic light saturation model (Eq. 1) for differences in maximum quantum yield of carbon dioxide (CO_2_) assimilation (α), maximum photosynthesis (P_MAX_) and dark respiration (A0). Model 1 includes all PFTs given in Table S2 and management categories. Model 2 also includes chloroplyll a+b content. Model 3 shows the direct impact of management and it was applied only to the data for the three PFTs (sedges, forbs and combined mire and forest mosses) that occurred in all management categories, in order to explore also the interaction between PFT and management category.

|  | P_MAX_ | | | | A0 | | | | α | | | |
| --- | --- | --- | --- | --- | --- | --- | --- | --- | --- | --- | --- | --- |
| **Model 1** | n DF | d DF | F-value | p-value | n DF | d DF | F-value | p-value | n DF | d DF | F-value | p-value |
| Intercept | 1 | 487 | 38.51 | <.0001 | 1 | 487 | 91.30 | <.0001 | 1 | 487 | 57.72 | <.0001 |
| PFT | 6 | 487 | 29.75 | <.0001 | 6 | 487 | 7.78 | <.0001 | 6 | 487 | 19.10 | <.0001 |
| Managem | 2 | 487 | 1.79 | 0.168 | 2 | 487 | 5.70 | 0.0036 | 2 | 487 | 1.74 | 0.176 |
| **Model 2** |  |  |  |  |  |  |  |  |  |  |  |  |
| Intercept | 1 | 484 | 6.12 | 0.0137 | 1 | 484 | 13.78 | 0.0002 | 1 | 484 | 8.42 | 0.0039 |
| PFT | 6 | 484 | 10.42 | <.0001 | 6 | 484 | 6.18 | <.0001 | 6 | 484 | 9.48 | <.0001 |
| Managem | 2 | 484 | 1.27 | 0.281 | 2 | 484 | 10.40 | <.0001 | 2 | 484 | 2.44 | 0.0881 |
| chl | 1 | 484 | 3.25 | 0.0721 | 1 | 484 | 9.88 | 0.0018 | 1 | 484 | 1.15 | 0.2844 |
| **Model 3** |  |  |  |  |  |  |  |  |  |  |  |  |
| Intercept | 1 | 385 | 26.96 | <.0001 | 1 | 385 | 163.72 | <.0001 | 1 | 385 | 38.19 | <.0001 |
| PFT2 | 2 | 385 | 39.30 | <.0001 | 2 | 385 | 44.46 | <.0001 | 2 | 385 | 30.49 | <.0001 |
| Managem | 2 | 385 | 6.03 | 0.0026 | 2 | 385 | 20.14 | <.0001 | 2 | 385 | 2.76 | 0.0643 |
| PFT2* Managem | 4 | 385 | 2.69 | 0.031 |  |  |  |  |  |  |  |  |
