# Supplementary material for: Vegetation structure and photosynthesis respond rapidly to restoration in young coastal fens
Source: Ecol Evol. 2016 Sep 7;6(19):6880–91. doi: 10.1002/ece3.2348 (PMC5513228; doi:10.1002/ece3.2348)
Supplement: Supplementary file 6 — Table S6. Impact of drainage and restoration on the light response of photosynthesis, Parameter estimates. [file ECE3-6-6880-s006.docx]

Table S6. Impact of drainage and restoration on the light response of photosynthesis. Parameter estimates from the hyperbolic light saturation model (Eq.2) based on nonlinear mixed-effects model fit, for Model 1, including 7 PFTs and management category and for Model 3 including 3 PFTs that occurred in all management categories, management category and their interaction. Undrained category and sedges are used as control.

|  |  | Pmax |  | A0 |  | α |  |
| --- | --- | --- | --- | --- | --- | --- | --- |
| **Model 1** |  |  |  |  |  |  |  |
| Source | DF | Value | Std.Error | Value | Std.Error | Value | Std.Error |
| **Fixed parameters** |  |  |  |  |  |  |  |
| Intercept | 487 | **273.9** | 44.1 | **-26.0** | 2.7 | **67.9** | 8.9 |
| Forb | 487 | **294.2** | 40.7 | **-12.2** | 3.3 | **80.2** | 11.3 |
| DecidShrub | 487 | 89.1 | 77.5 | 1.5 | 6.0 | **135.4** | 26.7 |
| EverShrub | 487 | **-118.0** | 57.5 | 2.8 | 4.4 | **221.7** | 52.6 |
| Grass | 487 | -5.4 | 53.1 | -0.2 | 4.3 | 6.1 | 14.0 |
| MireMoss | 487 | **-253.7** | 45.4 | **8.3** | 3.7 | **-38.1** | 13.9 |
| ForestMoss | 487 | **-277.1** | 46.5 | **13.8** | 3.8 | 20.5 | 18.0 |
| Restored | 487 | 53.0 | 56.4 | **-8.5** | 3.1 | 20.3 | 11.0 |
| Drained | 487 | 104.7 | 55.4 | **-9.5** | 3.0 | 6.2 | 10.3 |
| **Random parameters** |  | var(bks) |  | var(aks) |  | var(cks) |  |
| var |  | 173.5^2^ |  | 42.2^2^ |  | 9.5^2^ |  |
| cor (aks, bks) |  |  |  | -0.653 |  |  |  |
| cor (cks, bks) |  |  |  |  |  | 0.756 |  |
| cor (aks, cks) |  |  |  |  |  | -0.511 |  |
| **Model 3** |  |  |  |  |  |  |  |
| Source | DF | Value | Std.Error | Value | Std.Error | Value | Std.Error |
| **Fixed parameters** |  |  |  |  |  |  |  |
| Intercept | 385 | **236.98** | 45.64 | **-26.30** | 2.06 | **62.68** | 10.14 |
| Forb | 385 | **318.33** | 48.54 | **-10.04** | 2.43 | **81.40** | 12.30 |
| Moss | 385 | **-178.46** | 56.86 | **14.02** | 2.32 | -22.03 | 12.51 |
| Restored | 385 | 87.85 | 59.49 | **-9.62** | 2.37 | **26.63** | 12.38 |
| Drained | 385 | **217.22** | 62.75 | **-15.37** | 2.45 | 23.78 | 12.58 |
| Forb x restored | 385 | -44.11 | 53.37 |  |  |  |  |
| Moss x restored | 385 | -64.39 | 64.23 |  |  |  |  |
| Forb x drained | 385 | -64.44 | 58.13 |  |  |  |  |
| Moss x drained | 385 | **-182.21** | 60.36 |  |  |  |  |
| **Random parameters** |  | var(bks) |  | var(aks) |  | var(cks) |  |
| var |  | 178.6^2^ |  | 48.3^2^ |  | 4.7^2^ |  |
| cor (aks, bks) |  |  |  | -0.824 |  |  |  |
| cor (cks, bks) |  |  |  |  |  | 0.786 |  |
| cor (aks, cks) |  |  |  |  |  | -0.438 |  |
| Bolded values are significant with p<0.05 and underlined values have p<0.06)  var(e_{ksi})=11.3^2^ for model 1 and var(e_{ksi})=10.0^2^ for model 3. | | | | | | | |
