# Supplementary material for: Vegetation structure and photosynthesis respond rapidly to restoration in young coastal fens
Source: Ecol Evol. 2016 Sep 7;6(19):6880–91. doi: 10.1002/ece3.2348 (PMC5513228; doi:10.1002/ece3.2348)
Supplement: Supplementary file 7 — Table S7. Parameter estimates from the chlorophyll fluorescence (Fv/Fm) model. [file ECE3-6-6880-s007.docx]

Table S7. Parameter estimates from the Fv/Fm model (Eq.1) based on mixed-effects model fit, including 7 PFTs and management category. Undrained category and sedges are used as control.

| Source | DF | Value | Std.Error |
| --- | --- | --- | --- |
| Intercept | 616 | 0.758 | 0.02 |
| Forb | 616 | -0.002 | 0.00 |
| DecidShrub | 616 | **0.017** | 0.01 |
| EverShrub | 616 | **0.011** | 0.01 |
| Grass | 616 | **0.010** | 0.01 |
| MireMoss | 616 | **-0.092** | 0.03 |
| ForestMoss | 616 | -0.003 | 0.01 |
| Restored | 3 | 0.027 | 0.02 |
| Drained | 3 | 0.048 | 0.02 |
| Bolded values are significant with p<0.05 var(e_{ijk})=1451.519^2*exp(-var(e_{ijk})=1451.5^2^*exp(-2*13.4*ŷ) | | | |
